# Stanniocalcin-1 Regulates Re-Epithelialization in Human Keratinocytes

**DOI:** 10.1371/journal.pone.0027094

**Published:** 2011-11-01

**Authors:** Bonnie H. Y. Yeung, Chris K. C. Wong

**Affiliations:** Department of Biology, Hong Kong Baptist University, Kowloon Tong, Hong Kong; University of Birmingham, United Kingdom

## Abstract

Stanniocalcin-1 (STC1), a glycoprotein hormone, is believed to be involved in various biological processes such as inflammation, oxidative responses and cell migration. Riding on these emerging evidences, we hypothesized that STC1 may participate in the re-epithelialization during wound healing. Re-epithelialization is a critical step that involves keratinocyte lamellipodia (e-lam) formation, followed by cell migration. In this study, staurosporine (STS) treatment induced human keratinocyte (HaCaT) e-lam formation on fibronectin matrix and migration via the activation of focal adhesion kinase (FAK), the surge of intracellular calcium level [Ca^2+^]i and the inactivation of Akt. In accompanied with these migratory features, a time- and dose-dependent increase in STC1 expression was detected. STC1 gene expression was found not the downstream target of FAK-signaling as illustrated by FAK inhibition using PF573228. The reduction of [Ca^2+^]i by BAPTA/AM blocked the STS-mediated keratinocyte migration and *STC1* gene expression. Alternatively the increase of [Ca^2+^]i by ionomycin exerted promotional effect on STS-induced *STC1* gene expression. The inhibition of Akt by SH6 and GSK3β by lithium chloride (LiCl) could respectively induce and inhibit the STS-mediated e-lam formation, cell migration and *STC1* gene expression. The STS-mediated e-lam formation and cell migration were notably hindered or induced respectively by STC1 knockdown or overexpression. This notion was further supported by the scratched wound assay. Collectively the findings provide the first evidence that STC1 promotes re-epithelialization in wound healing.

## Introduction

Human stanniocalcin-1 (STC1) is a glycoprotein hormone that is widely expressed in various tissues [1], [2]. Modulation of STC1 expression has been reported in numerous physiological and pathological processes, such as cell proliferation/apoptosis [3], [4], [5], inflammation [6], [7], angiogenesis [8], [9] and steroidogenesis [10]. Emerging evidences have pointed out the involvement of STC1 in carcinogenesis [11], [12], [13], [14], [15], [16], [17], [18], [19]. It is generally believed that both carcinogenesis and wound healing show similar biological features in the processes of inflammation and angiogenesis [20]. At the molecular level, notable similarities in gene expression between cancers and wounds have been reported [21]. Based on the previous findings of STC1 on carcinogenesis, we hypothesized that STC1 may take part in the wound healing process.

Wound healing plays a vital role for the maintenance of the integrity of the skin and mucosal membranes. In fact, there are three major skin responses after injury, including inflammation, re-epithelialization (migration of keratinocytes) and remodeling (formation of granulation tissues) [22]. To maintain the normal healing process, the presence of both macrophages and T lymphocytes at the wound bed is essential [23]. In a cell migration study, the transendothelial migration of human umbilical vein endothelial cells was found to be inhibited by STC1 [6]. In contrast to the inhibitory effect of STC1 on the transendothelial migration, STC1 exerted a promigratory effect on human ovarian cancer cells [14]. Another study however reported a selective modulatory role of STC1 on hepatocyte growth factor-induced endothelial migration [9]. Riding on the inconclusive role of STC1 on the cell migration process, we are interested in elucidating the regulation and function of STC1 in keratinocyte migration, a critical step for tissue repair and wound healing.

Staurosporine (STS) a broad kinase inhibitor confers a useful tool to study cell migration as STS-induced aggressive phenotypes have been illustrated in a variety of cell types [24], [25], [26]. Particularly, treatment of STS in human normal epidermal keratinocyte cell line HaCaT could sufficiently induce lamellipodia extension (e-lam) and cell migration [27]. In this study, we attempted to elucidate the role of STC1 in keratinocyte re-epithelialization during the wound healing process. Our data have demonstrated the enhancing effect of STC1 on STS-stimulated e-lam formation and cell migration via Akt pathway.

## Results

### Staurosporine induces focal adhesion kinase phosphorylation, formation of extended lamellipodia on fibronectin matrix, cell motility and STC1 mRNA expression

**Figure 1:**
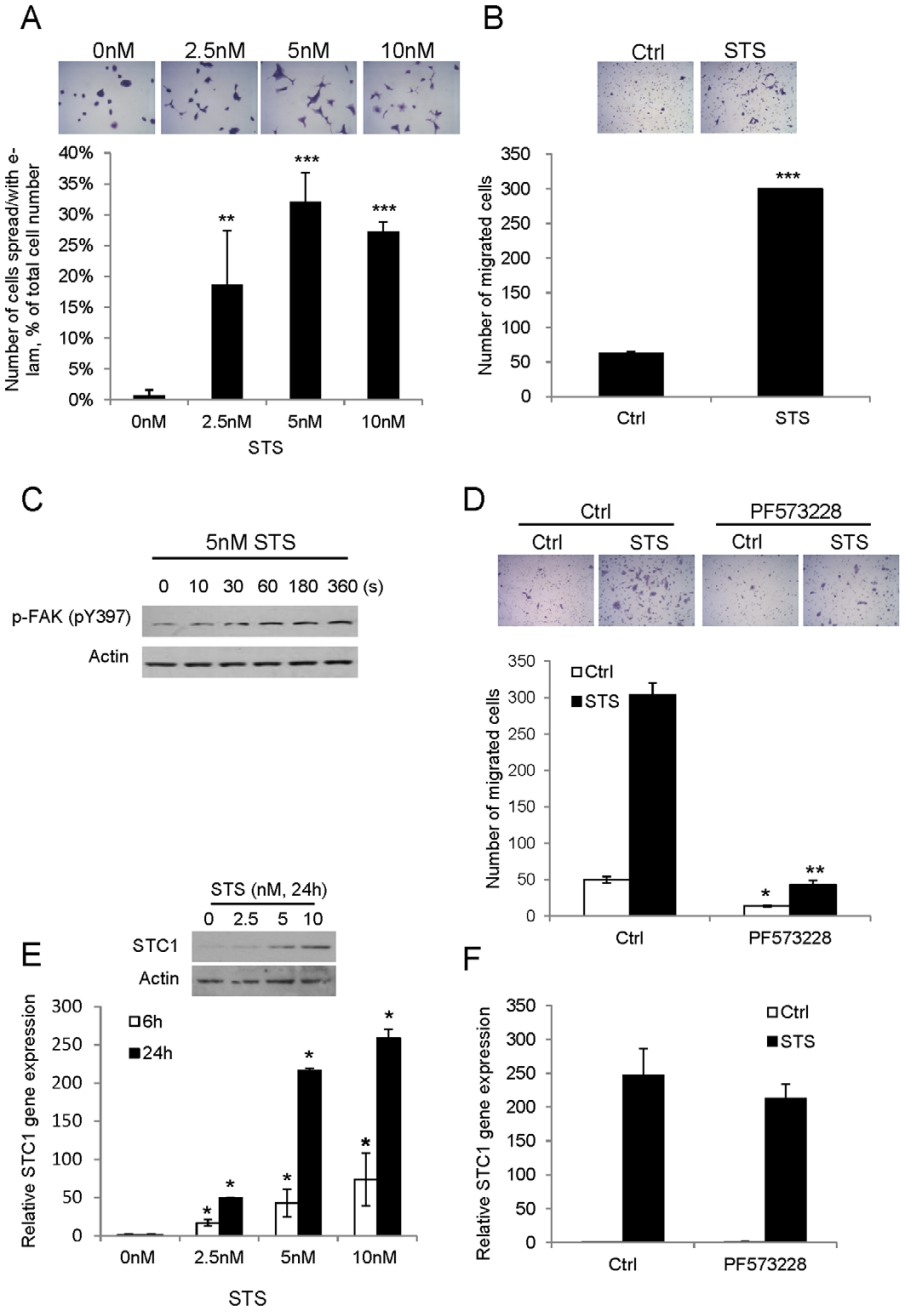
STS induces keratinocyte migration and *STC1* mRNA expression. (A) In the cell spreading assay, e-lam formation on fibronectin-coated plate was induced by increasing doses of STS treatment (0, 2.5, 5, 10 nM) for 24 h. The cell images were captured under magnification of 200x (*top*). (B) Cell migration through the transwell was induced by 5 nM STS for 24 h and the cell images were captured under 100x magnification (*top*). (C) Western blot analysis demonstrated the increase of FAK phosphorylation (pY397) upon 5 nM STS treatment from 10–360 s. (D) Cell migration was increased upon STS treatment at 24 h, but was blocked by 2 µM PF573228. Cell images were captured under 100x magnification (*top*). (E) *STC1* mRNA and protein expressions were induced by increasing doses of STS treatment (0, 2.5, 5, 10 nM). (F) STS-induced *STC1* mRNA expression was not significantly abolished by PF573228 at 24 h. Asterisks (***) denote *p*<0.0001, (**) denote *p*<0.005 and (*) denote *p*<0.01 as compared to the respective control treatment.

Extended lamellipodia (e-lam) means lamellipodia containing a growth cone at the tip. The formation of e-lam on fibronectin matrix and the increase of keratinocyte migration are the two critical steps occurred during wound healing [28], where focal adhesion kinase (FAK) autophosphorylation at Tyr-397 is one of the key molecules in fibronectin-stimulated signaling to stimulate cell motility [29]. Upon stimulation of the HaCaT cells by STS for 24 h, the number of e-lam formation on the fibronectin-coated plate was dose-dependently increased, but wasn’t at the highest dose of treatment (10 nM) owing to a significant increase in STS-induced cell death (Fig 1A). By using Boyden Chamber, STS treatment (5 nM) increased cell motility by 5-fold at 24 h as compared with the control (Fig.1B). The STS treatment also stimulated FAK phosphorylation at Tyr-397 (Fig. 1C). Using FAK inhibitor (PF573228), the degree of keratinocyte migration in both control and STS treated cells were suppressed (3.64- and 7.11-fold reduction in the Ctrl and STS treatment, respectively, Fig. 1D). The STS treatment was also able to upregulate *STC1* mRNA and protein expressions in a dose-dependent manner (2.5–10 nM) (Fig. 1E). Intriguingly STS-induced STC1 expression could not be significantly inhibited by PF573228 c-treatment (Fig. 1F).

### Staurosporine-induced events are mediated by an increase of intracellular free Ca^2+^

**Figure 2:**
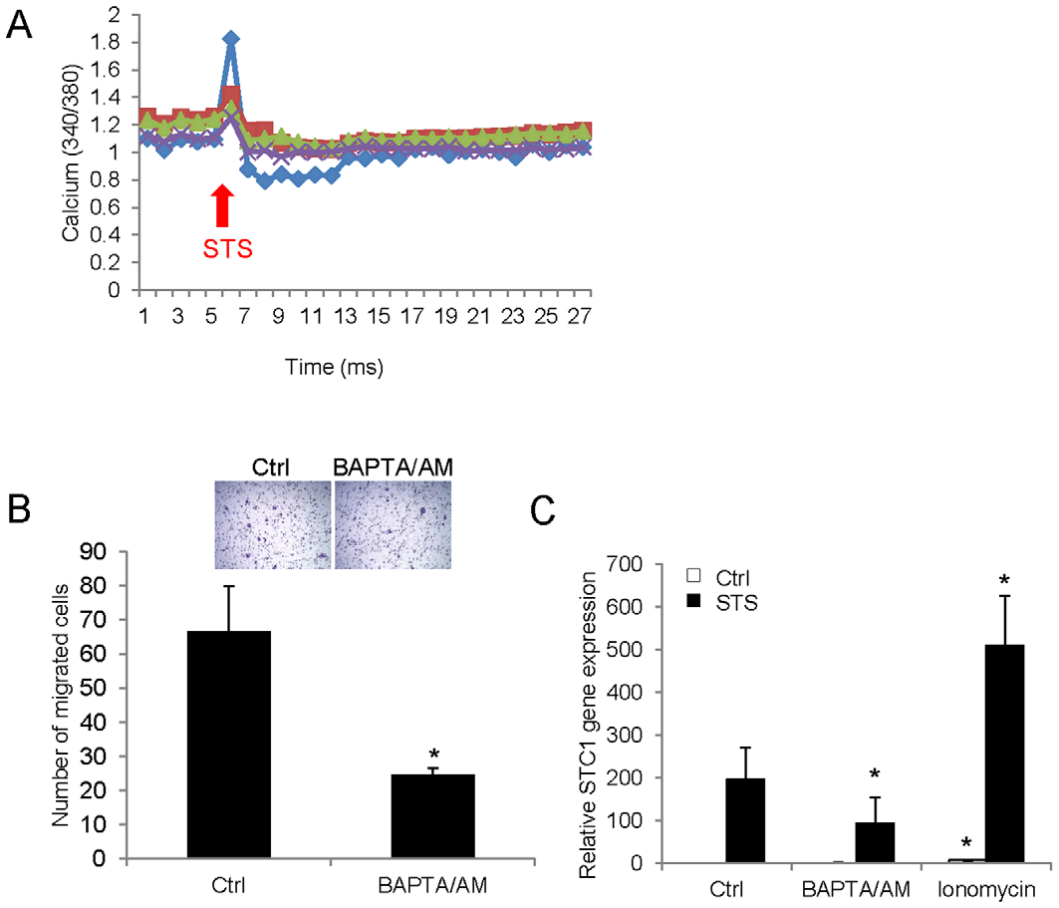
STS-induced migration is mediated by intracellular Ca^2+^ influx. (A) STS treatment increased [Ca^2+^]i as measured using Ca^2+^ detection dye, Fura 2-AM. (B) The number of migrated cells was significantly reduced by 10 µM BAPTA/AM at 24 h (vs Ctrl) and the cell images were captured under 100x magnification (*top*). (C) STS-induced *STC1* mRNA was further induced by 1 µM ionomycin but was downregulated by 10 µM BAPTA/AM at 24 h of the cotreatments. Asterisks (*) denote *p*<0.05 as compared to the Ctrl.

By measuring intracellular Ca^2+^ [Ca^2+^]i using Fura 2-AM, an increase of [Ca^2+^]i was demonstrated in the STS treated cells. The ratio 340 nm/380 nm was surged immediately after an addition of 5 nM STS, which indicated that STS was able to elevate [Ca^2+^]i in the HaCaT cells (Fig. 2A). When an intracellular calcium chelator BAPTA/AM, was added into the cells, cell motility was suppressed by 2.77-fold (Fig. 2B). To ascertain the effect of [Ca^2+^]i on STC1 expression, both BAPTA/AM and the potent calcium ionophore agent ionomycin, were used to pretreat the cells for 1 h before STS stimulation. In the absence of STS, *STC1* mRNA showed an 8-fold induction in 1 µM ionomycin treatment. No noticeable effect was observed in BAPTA/AM alone treatment (Fig. 2C). In the presence of STS, *STC1* mRNA expression was synergistically enhanced by ionomycin (2.53-fold induction versus STS alone), but was reduced by 2.52-fold upon BAPTA/AM pretreatment (Fig. 2C).

### Staurosporine-induced events are mediated by Akt-GSK3β signaling

**Figure 3:**
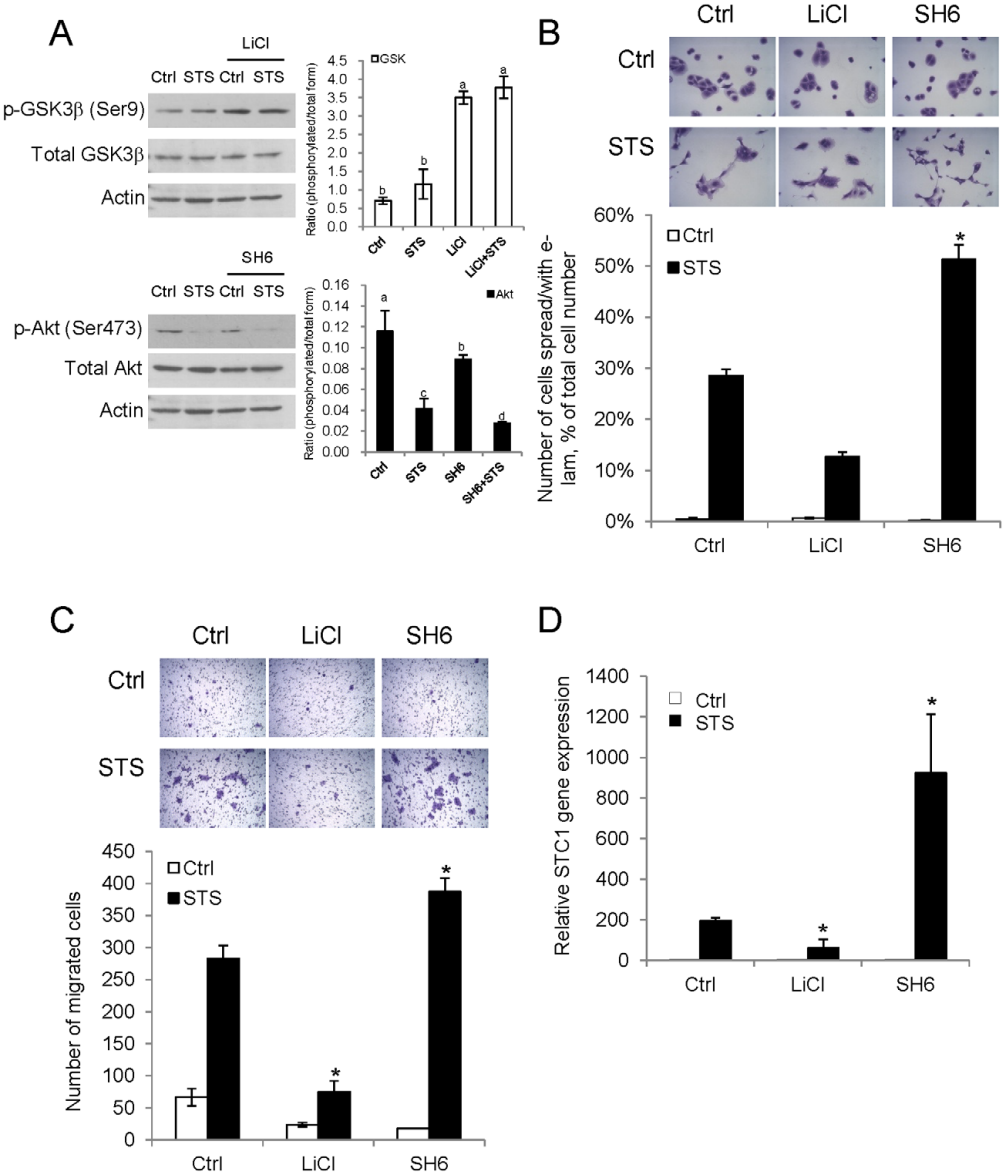
Akt-GSK3β signaling mediates the keratinocyte migration process. (A) The inhibitory effects of 30 mM LiCl and 10 µM SH6 on GSK3β and Akt-signaling respectively, were determined by probing the phosphorylated and total forms of GSK3β and Akt. The band intensities were measured and the ratios of phosphorylated/total form were plotted. Bars with the same letter are not significantly different according to the results of one-way ANOVA followed by Duncan’s multiple range tests (*p*<0.05). STS-induced (B) e-lam formation and (C) cell migration were suppressed by LiCl but were induced by SH6. The cell images were captured under 200x and 100x magnification for e-lam formation and cell migration, respectively (*top*). (D) STS-induced *STC1* mRNA expression was inhibited by LiCl, but was increased by SH6 (vs STS alone). Asterisks (*) denote *p*<0.05 as compared to their corresponding control.

Koivisto’s group demonstrated that e-lam formation in the HaCaT cells is regulated by GSK3β-signaling pathway [27]. In the fact that GSK3β and Akt are tightly coordinated [30], studying the role of Akt-GSK3β in our model was necessary. Using pharmacological approaches, LiCl and SH6 were used respectively, to inhibit GSK3β and Akt pathways. The cells were pretreated with 30 mM LiCl or 10 µM SH6 for 1 h before the addition of STS. In Fig. 3A, the protein lysates were extracted after treatment and were probed for both the phosphorylated and total forms of GSK3β and Akt. Activation of GSK3β was depicted by the de-phosphorylation of GSK3β (Ser9); whereas the activation of Akt was indicated by the phosphorylation at Ser473. Figure 3A showed the inhibitory effects of LiCl and SH6 on GSK3β and Akt respectively. STS treatment could significantly suppress Akt phosphorylation but not on GSK3β. STS elicited e-lam formation was blocked by LiCl (2.25-fold reduction versus STS alone), but augmented in SH6 pretreatment (1.8-fold induction versus STS alone) (Fig. 3B). Using the cell migration assay, STS-mediated migration was suppressed upon LiCl pretreatment (3.77-fold versus STS alone), but was stimulated in SH6-pretreated cells (1.37-fold versus STS alone) (Fig. 3C). Likewise, STS-induced *STC1* mRNA expression was inhibited by 3.8-fold and augmented by 2.6-fold (versus STS alone) in LiCl and SH6 pretreatment, respectively (Fig. 3D).

### STC1 knockdown hinders STS-induced extended lamellipodia and cell migration

**Figure 4:**
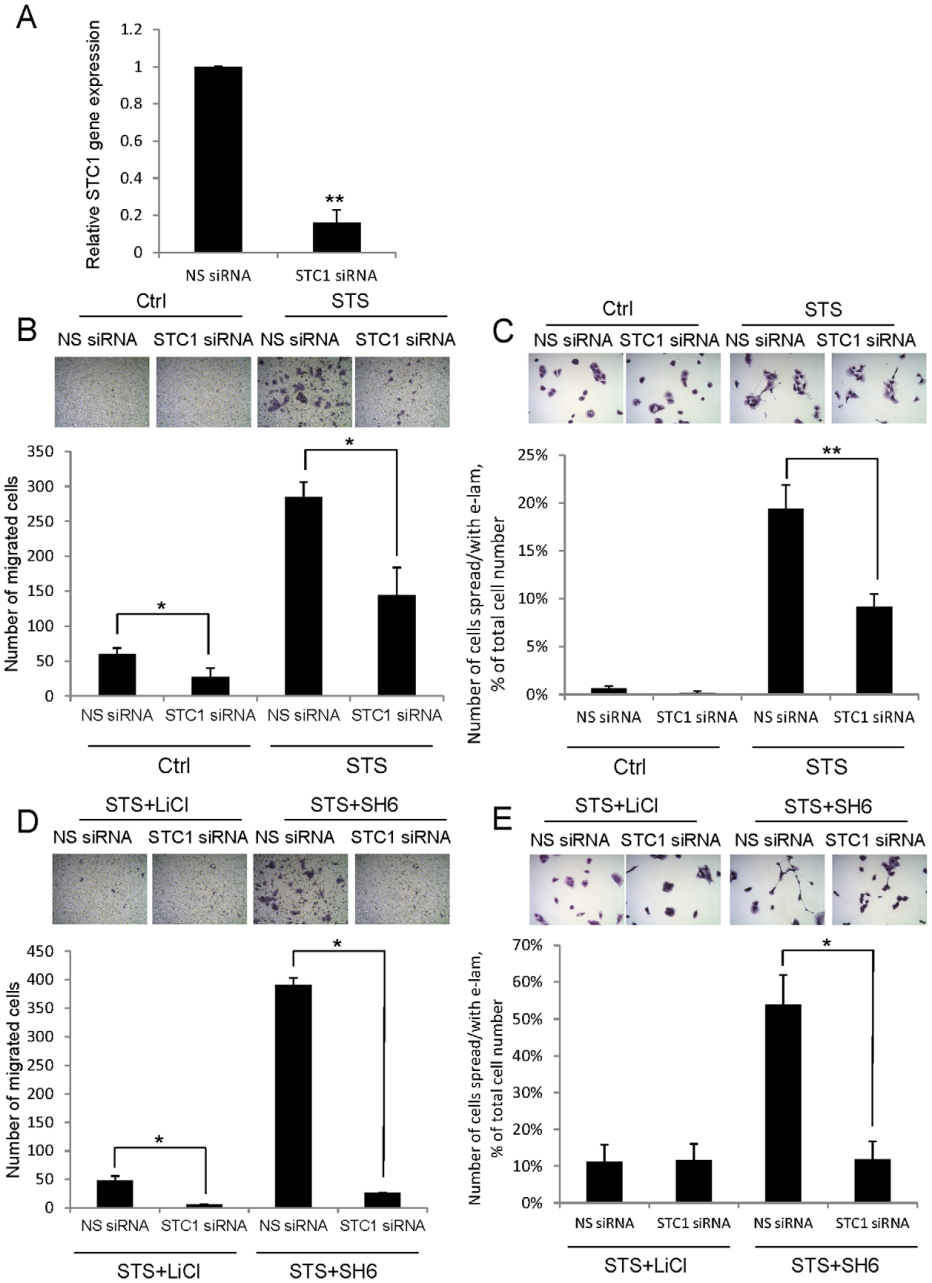
STC1 knockdown hinders STS-mediated re-epithelialization process. (A) After the transfection of STC1 siRNA for 48 h, the knockdown efficiency was examined by real-time PCR. In STC1 siRNA-transfected cells, STS-induced (B) cell migration and (C) e-lam formation on the fibronectin-coated plates were significantly reduced as compared to the NS siRNA-transfected cells. The cell images were captured for e-lam formation (200x magnification) and cell migration (100x magnification) (*top*). Under the STS+LiCl or STS+SH6 cotreatment, (D) the number of migrated cells and (E) e-lam formation on fibronectin-coated plate were compared between the STC1 siRNA-transfected cells and the NS siRNA-transfected cells. The knockdown of STC1 was found to inhibit cell migration induced by the STS + LiCl/SH6 cotreatments. The synergistic effect of STS/SH6 on the increase of e-lam formation was significantly reduced in the STC1 siRNA transfected cells. Asterisks (**) denote *p*<0.01 and (*) denote *p*<0.05 as compared to their corresponding NS siRNA-transfected cells.

We then conducted STC1 knockdown using siRNA technique, to reveal its role in STS-mediated re-epithelialization. After 48 h post-transfection of STC1 siRNA in the HaCaT cells, STC1 gene expression level was remarkably reduced by 6.29-fold as compared with the NS siRNA-transfected cells (Fig. 4A). Strikingly in STC1 siRNA-transfected cells, 2.13- and 1.98-fold reduction in STS-mediated cell migration (Fig. 4B) and e-lam formation (Fig. 4C) were detected. In the STS and LiCl/SH6 cotreatment, the knockdown STC1 was found to inhibit cell migration (Fig. 4D). The synergistic effect of STS/SH6 on the increase of e-lam formation was also significantly reduced in the STC1 siRNA transfected cells (Fig. 4E).

### STC1 overexpression promotes STS-induced extended lamellipodia and cell migration

**Figure 5:**
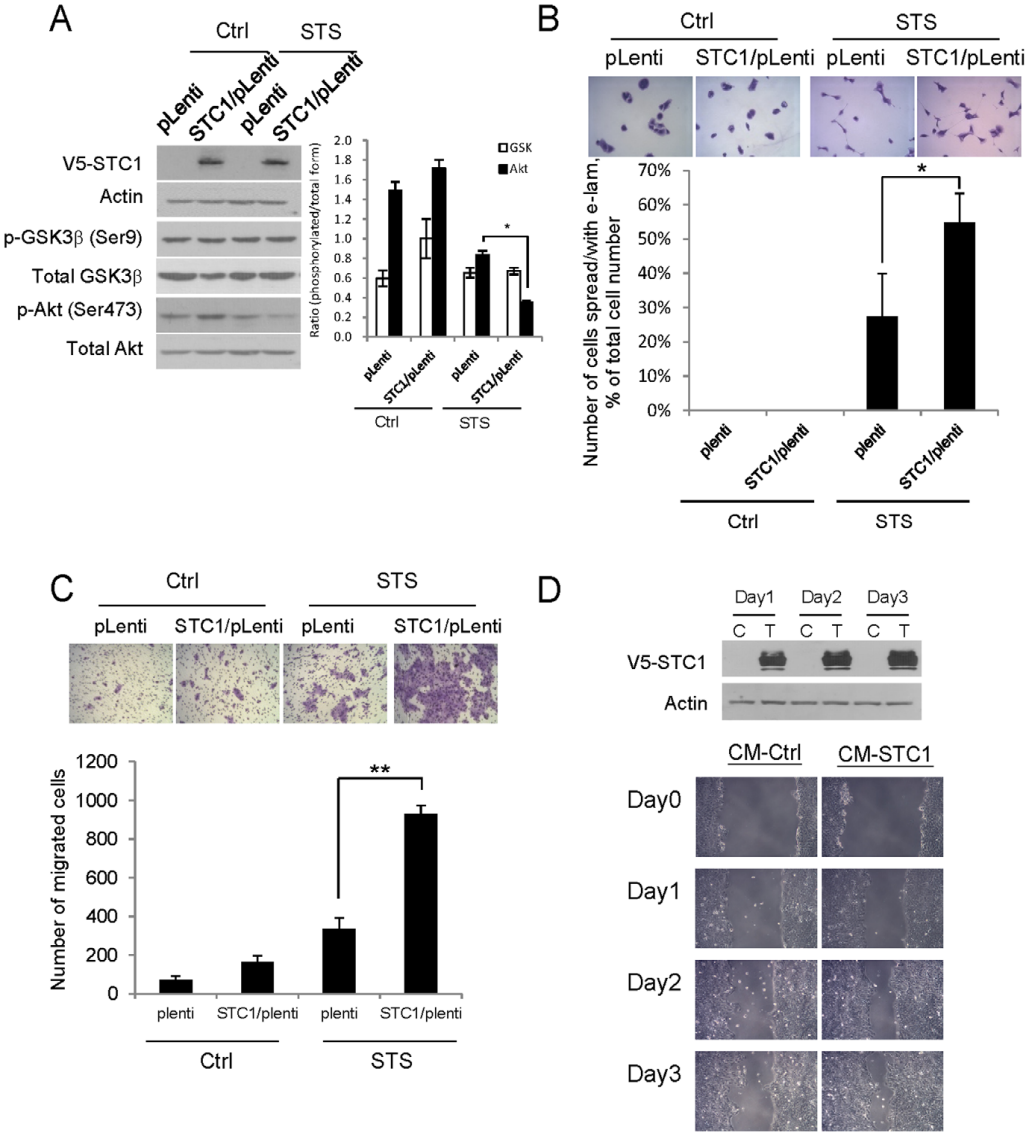
STC1 enhances STS-mediated re-epithelialization process. (A) After transient transfection of V5-tagged STC1/pLenti (STC1/pLenti) and empty vector control (pLenti) into the HaCaT for 24 h, the cells were treated with 5 nM STS for 24 h and the protein expression levels of V5, GSK3β and Akt were determined using western blotting. The band intensities were measured and the ratios of the respective phosphorylated/total proteins were plotted. STC1 overexpression was found to have synergistic effect on STS-inhibited phosphorylation of Akt. STS-induced (B) e-lam formation on fibronectin-coated plate and (C) cell migration were synergistically induced by the transient overexpression of STC1 (vs STS-treated pLenti). The cell images were captured for e-lam formation (200x magnification) and cell migration (100x magnification) (*top*). (D) In the scratched wound healing assay, the scratched wound was closed significantly more rapidly in the cells maintained in the conditioned medium (CM) containing overexpressed STC1 protein (CM-STC1) than the control medium (CM Ctrl). The cell images were captured under 100x magnification. The levels of STC1 proteins in the conditioned media (CM-Ctrl and CM-STC1) were determined from day1 to day3 using western blotting. (*top*); CM-Ctrl depicts as C and CM-STC1 depicts as T. Asterisks (**) denote *p*<0.01 and (*) denote *p*<0.05 as compared to STS-treated pLenti-transfected cells.

In addition to STC1 knockdown, the effects of transient overexpression of STC1 on STS-induced e-lam and cell migration were investigated. In Fig. 5A, the overexpression of V5-tagged STC1 in the HaCaT cells was confirmed using V5 antibody. In the presence of STS, the overexpression of STC1 (STC1/pLenti) in STS-treated cells could further suppress Akt activation as compared with the empty vector transfected cells (pLenti). The overexpression however had no significant effect on the GSK3β activity. In the unstimulated cells, the overexpression of STC1 alone was not able to promote e-lam formation (Fig. 5B) and cell migration (Fig. 5C). However in the STS-stimulated cells, STC1 overexpression was able to augment both e-lam formation (1.9-fold) and cell migration (2.79-fold). Moreover STC1 overexpression was not able to avert the inhibitory effect of LiCl on e-lam formation and cell migration (data not shown). Nevertheless the pro-migratory effect of STC1 was further supported by the data of the scratched wound healing assay. Using the conditioned medium (CM) prepared from the established tetracycline-inducible STC1-expressing CNE2 cells, the scratched wound was found to be rapidly closed in the HaCaT cells maintained in the conditioned medium containing overexpressed STC1 protein (CM-STC1) (Fig. 5D). Both cell count and MTT assays suggested that there was no significant difference in the rate of cell proliferation between the HaCaT cells incubated in the condition media with (CM-STC1) or without overexpressed STC1 (CM-Ctrl) (data not shown).

## Discussion

STC1 has been reported to be involved in a variety of physiological/pathological processes including inflammation, angiogenesis and oxidative stress [6], [7], [9], [31], [32], [33], [34], [35], [36], [37], [38], [39], and these findings prompted us to investigate whether STC1 plays role in wound healing process. Re-epithelialization is a critical step for wound healing process that involves migration and proliferation of keratinocytes [28]. Keratinocyte migration comprises of a repeated cycle of protrusion, adhesion, cell contraction, and rear release [40]. Protrusion, the formation of e-lam, is a key step for the establishment of cell polarity to initiate migration. Therefore in this study, we attempted to investigate the role of STC1 on the process of re-epithelialization. Using the human keratinocyte model HaCaT cells, we were able to illustrate the involvement of STC1 in re-epithelialization especially in e-lam formation and keratinocyte migration. The process of re-epithelialization and STC1 expression are found to be regulated by Ca^2+^ and Akt-GSK3β signaling.

In this model, keratinocyte e-lam formation and migration were found to be associated with [Ca^2+^]i elevation and focal adhesion kinase (FAK) activation. This agrees with the recent findings that an increase of [Ca^2+^]i was detected in STS stimulation of cell migration [41], [42]. In particularly, STS-induced Ca^2+^ mobilization is necessary for the activation of calcium-dependent enzymes in the wound sites [43] to initiate cell motility [42] and to facilitate rear-end detachment [44]. In accompanied with the activation of FAK and Ca^2+^-signaling, *STC1* mRNA expression was found to be upregulated. To decipher the regulatory role of FAK/Ca^2+^-signaling on the induction of STC1 expression, the inhibitors and/or the activator of the respective pathway were assessed. Surprisingly, STS-induced STC1 expression was found to be independent of FAK signaling, although the possible regulatory relationship was suggested [9]. Moreover the positive regulatory role of Ca^2+^ on STC1 expression was found to be consistent in respect to the conserved biological functions of STC1 on calcium regulation in vertebrates [6], [45], [46], [47]. In this study the involvement of STC1 on Ca^2+^-stimulated keratinocyte migration is firstly revealed. In addition to Ca^2+^-signaling, the pro-migratory effect of STC1 in the STS-treated cells was, at least in part via the suppression of Akt pathway. Akt signaling involves in a wide range of cellular processes such as proliferation, differentiation, migration and invasion [48]. Using a pharmacological approach, both re-epithelialization process and *STC1* expression were augmented in response to Akt inhibition, but *vice versa* under GSK3β inhibition. The data are consistent with other study in which Akt-mediated GSK3β inhibition showed an abrogate effect on cell migration in intestinal epithelium cells [49]. Intriguingly STS-induced *STC1* gene expression was accompanied with Akt inhibition, but had no noticeable effect on the activation of GSK3β. The data imply that other downstream targets of Akt pathway, like mTOR, FOXO, IκB kinase (IKK) and Mdm2 [48] might also be involved in *STC1* gene regulation. For instance, activation of Akt could increase NF-κB activity via phosphorylation of IKK [50], while the negative effect of NF-κB on STC1 expression was reported in the mouse neuroblastoma cell model [51]. In addition, Akt could inhibit p53 via phosphorylation of Mdm2 [52], wherein p53 could stimulate STC1 expression in human nasopharyngeal cancer cells [3].

In the STC1 knockdown and overexpression experiments, the pro-migratory effects of STC1 on STS-induced cells were demonstrated. However STC1 overexpression in the un-stimulated cells failed to induce e-lam formation and cell migration. In considering the inconclusive observations on the role of STC1 in cell migration process [6], [9], [14], our data imply that STC1 may not be a key regulator for or against this process. The particular role of STC1 is probably controlled in cells under different settings that regulate the pro- or anti-migratory process. From the results of the STS and LiCl/SH6 co-treatment in the STC1 siRNA transfected cells, the data support the notion that STC1 is negatively regulated by Akt-signaling. Consistently the STC1 overexpression experiments illustrate the negative regulatory role of STC1 on Akt pathway. Retrospectively the activation of GSK3β signaling could stimulate STC1 expression and enhance the pro-migratory process.

In conclusion, STS treatment stimulated the transient increase in FAK phosphorylation and [Ca^2+^]i which inhibited Akt signaling pathway, to initiate re-epithelialization in the HaCaT cells. The activation of GSK3β and some other Akt-related signaling molecules stimulate STC1 expression and facilitate keratinocyte migration. Using both knockdown and overexpression approaches, the pro-migratory role of STC1 in STS-induced HaCaT cells are confirmed. Our data warrant further investigation on the regulation and function of STC1 in the tissue repair and wound healing process.

## Materials and Methods

### RNA and Protein Extraction, Real-time PCR and Western blotting

Total RNA was extracted by TRIZOL according to the manufacturer’s instructions (Invitrogen), and subjected to DNase I digestion using TURBO DNAfree (Ambion) to remove any genomic DNA contamination. Reverse transcription was carried out using HC RNA-cDNA Master Mix (Applied Biosystems). Real time-PCR was performed using Fast SYBR Green Master Mix (Applied Biosystems). The primer sequences for STC1 and β-actin were as followed: STC1 forward 5′-TGAGGCGGAGCAGA ATGACT-3′ and reverse 5′-CAGGTGGAGTTTTCCAGGCAT-3′, and β-actin forward 5′-GACTACCTCATGAAGATCCTCACC-3′ and reverse 5′-TCTCCTTAA TGTCACGCACGATT-3′. The amplification cycles were 95°C for 1 min, followed by 40 cycles of 95°C for 10 s, 56°C for 10 s and 72°C for 30 s using the ABI StepOne Real-time PCR System (Applied Biosystems).

For western blotting, protein lysates were extracted from the cells using RIPA buffer (50 mM Tris-HCl, pH 7.4, 150 mM NaCl, 2 mM EDTA, 1% NP-40, 0.1% SDS) and the protein concentrations were determined by DC protein assay kit II (Bio-Rad). The protein lysates were resolved by SDS-PAGE and were blotted as described previously [34]. The blotting was conducted using mouse anti-V5 (Invitrogen), anti-human STC1 (R&D), human p-FAK (pY397) (BD Biosciences), rabbit anti-human p-GSK3β (Ser9), anti-human total GSK3β, anti-human p-Akt (Ser473), anti-human total Akt (Cell Signaling), and anti-human actin serum (Sigma). Protein bands were visualized by enhanced chemiluminescence system (WESTSAVE Up, LabFrontier).

### Cell culture

Human normal epidermal keratinocyte cell line HaCaT was purchased from ATCC. HaCaT is a spontaneously immortalized keratinocyte cell line, which maintains most of properties of normal epidermal keratinocytes [53]. The cells were cultured in Dulbecco’s modified Eagle’s medium (low glucose) (Invitrogen) supplemented with antibiotics (50 U/ml penicillin and 50 µg/ml streptomycin) (Invitrogen) and 10% heat-inactivated fetal bovine serum (FBS) (HyClone®, Perbio) and were seeded overnight before treatment or transfection. Human nasopharyngeal carcinoma cell line CNE2 was a gift from Prof. NK Mak, Hong Kong Baptist University, Hong Kong. The CNE cells were cultured in RPMI 1640 medium (Invitrogen) supplemented with antibiotics (50 U/ml penicillin and 50 µg/ml streptomycin) and 10% FBS. Human embryonic kidney cell line HEK293FT (Invitrogen) was cultured in Dulbecco’s modified Eagle’s medium (high glucose) (Invitrogen) supplemented with antibiotics (50 U/ml penicillin and 50 µg/ml streptomycin), 10% FBS, 6 mM L-glutamine (Sigma), 1 mM sodium pyruvate (Sigma). The drugs, staurosporine (STS), BAPTA/AM, ionomycin and SH6 were purchased from Calbiochem, and lithium chloride (LiCl) was purchased from Sigma-Aldrich, and PF573228 was purchased from Torcis. In the study of the drugs mediated events following STS treatment, the cells were pretreated with the drugs for 1 h before the addition of STS.

### Small interfering RNA (siRNA) transfection

ON-TARGETplus SMARTpool targeting human *STC1* (STC1 siRNA) and a non-targeting control (NS siRNA) were purchased from Dharmacon. The target sequences of STC1 siRNA were: 5′-AAACGCACAUCCCAUGAGA-3′; 5′-GGGAAAAGCAUUCGUCAAA-3′; 5′-GUACAGCGCUGCUAAAUUU-3′ and 5′-CAACAGAUACUAUAACAGA-3′. The target sequence of NS siRNA was 5′-GGCUACGUCCAGGAGCGCA-3′. Briefly, HaCaT cells (7×10^4^) were seeded in the 12-well plates overnight. One hundred µl of 50 nM siRNA and 1.5 µl of siLentFect (Bio-Rad) complex was mixed and was added into the cells according to the manufacturer’s instruction. The efficiency of STC1 knockdown was examined at 48 h of post-transfection using real-time PCR.

### Overexpression of STC1

#### Construction of pLenti6.3/TO/V5-DEST-STC1 plasmid

A human *STC1* cDNA encoding wild-type full-length without the stop codon was amplified by PCR and was cloned into pENTR™/SD/D-TOPO (Invitrogen) according to the manufacturer’s instruction. Then the STC1 insert was transferred from pENTR™/SD/D-TOPO into an expression vector pLenti6.3/TO/V5/-DEST (Invitrogen) using Gateway® LR Clonase™ II Plus Enzyme Mix (Invitrogen).

#### Transient transfection of STC1 in the HaCaT cells

pLenti6.3/TO/V5-DEST-STC1 (STC1/pLenti) or pLenti6.3/TO/V5 (pLenti) was transfected into the HaCaT cells using Lipofectamine 2000 (Invitrogen) according to the manufacturer’s instruction. Protein expression level of STC1 was confirmed at 24 h of post-transfection using western blot.

#### Lentivirus overexpression of STC1 in the CNE2 cells

Tetracycline-inducible STC1 overexpression in the CNE2 cells was established using ViraPower™ HiPerform™ T-REx™ Gateway® Vector Kit (Invitrogen) and was conducted as described in the manufacturer’s instruction. Briefly, 80% confluence of HEK293FT cells were seeded on 100 mm cell culture dishes overnight. Lentivirus was produced by the cotransfection of ViraPower™ Packaging mix (Invitrogen) with either the pLenti6.3/TO/V5-DEST-STC1 (STC1/pLenti) for STC1 expression or pLenti3.3/TR (Invitrogen) for tetracycline (Tet) repressor expression using Lipofectamine 2000. Viral supernatants were harvested at 48 h after transfection. The viral supernatant was ready-to-use or can be stored at −80°C. CNE2 cells (1×10^5^) were seeded in 6-well plates overnight, and were then transduced with 1 ml of pLenti3.3/TR lentiviral particles with 6 µg/ml of polybrene® (Sigma). After 24 h of incubation, the medium containing pLenti3.3/TR lentiviral particles was removed and was replaced by 1 ml of STC1/pLenti lentiviral particles with 6 µg/ml of polybrene® and was incubated for another 24 h. In brief, the stable colonies of CNE2 cells that expressed STC1 and Tet repressor were selected in RPMI 1640 medium containing 4 µg/ml blasticidin (Invitrogen) and 600 µg/ml geneticin (Sigma). STC1 overexpression in CNE2 cells was induced by an addition of 1 µg/ml of tetracycline (Invitrogen) for 24 h. The expression level of STC1 protein in the conditioned media (CM) was checked by western blotting.

### Boyden chamber-based cell migration assay

Cell migration was performed using 24-well Transwell inserts with 8 µm pore size membrane (Costar, Corning). HaCaT cells were trypsinized and were washed with serum-free medium twice. Then, the cells (6×10^4^) were seeded into the upper chamber of the insert. The complete medium as a chemo-attractant, was added into the lower chamber. After 24 h incubation at 37°C, the cells on the top of the membrane were removed by cotton swabs and the cells on the bottom (migrated cells) were rinsed with PBS, fixed in methanol followed by staining with 0.5% crystal violet (Farco Chemical Supplies). The total number of migrated cells was counted under microscope and cell morphology was captured at 100× magnification.

### Scratch wound healing assay

Wound healing assay was conducted using 6-well culture plates. The HaCaT cells were seeded to a confluent density and were wounded by dragging a plastic pipette tip. The cells were then incubated in the conditioned media with or without STC1 overexpressed protein. Cell images were captured from day 1 to day 3 under an inverted microscope (100× magnification). To elucidate if the restoration of the scratch wound was due to an increase of proliferative rate of the cells, cell counting and MTT assays were conducted. HaCaT cells (5×10^4^) were seeded in 12-well plates overnight, in the conditioned medium with or without STC1 overexpressed protein. On day1–3, the cells were trypsinized and were mixed 1∶1 with trypan blue solution. The number of cell was counted using hemocytometer using a microscope (AE31, Motic). Cell viability was also assessed by MTT [3-(4,5-dimethyl-thiazol-2-yl)-2,5- diphenyltetrazolium bromide] (Sigma-Aldrich) assay. HaCaT cells (8×10^3^) were seeded in 96-well plates in the conditioned medium with or without STC1 overexpressed protein for 3 days. On each day, the cells were incubated with 500 µg/ml of MTT at 37°C for 4 h. The medium was then aspirated, followed by resuspension in 100 µl of DMSO. The absorbance was measured at 540 nm by a plate reader (ELx800, BioTek).

### Cell spreading assay

Before cell seeding, 24-well culture plates were coated with 5 µg/ml of fibronectin (Millipore) and were kept at 4°C overnight, followed by blocking with 1% bovine serum albumin (BSA, USB) for 30 min at room temperature. Then, HaCaT cells (2×10^4^) cells were seeded and allowed to spread for 24 h. The cells were then fixed with Cytoskelfix™ Cell Fixative (Cytoskeleton) at −20°C for 10 min followed by staining in 0.5% of crystal violet. The cell images were captured at 200x magnification in at least 5 random fields in each well and the percentage of cells spread with e-lam was counted.

### Intracellular fluorescence measurement of calcium

Intracellular calcium level was measured by using a dual-excitation fluorescence photomultiplier system (Nikon). HaCaT cells (1×10^5^) were seeded on sterilized glass coverslips in 6-well culture plates overnight, followed by incubation in 1 µM of Fura 2-AM dye (Calbiochem) at 37°C for 2 h. The coverslip was mounted in a custom-designed device and was placed on the stage of the microscope. Fura 2-AM fluorescence (emission: 510 nm) following alternate excitation at 340 and 380 nm was continuously monitored before and after the addition of 5 nM STS. The fluorescence ratio (340/380 nm) and images were acquired using MetaFluor software (Universal Imaging).

### Statistical analysis

All data were analyzed by a Student’s *t*-test or one-way analysis of variance (ANOVA) followed by Duncan’s multiple range test. All data are represented as statistical means ± SD. A *p* value <0.05 was considered statistically significant. All experiments were performed in triplicates at least.
